# Genetic implications of bottleneck effects of differing severities on genetic diversity in naturally recovering populations: An example from Hawaiian coot and Hawaiian gallinule

**DOI:** 10.1002/ece3.3530

**Published:** 2017-10-20

**Authors:** Sarah A. Sonsthagen, Robert E. Wilson, Jared G. Underwood

**Affiliations:** ^1^ Alaska Science Center U.S. Geological Survey Anchorage AK USA; ^2^ Pacific Reefs National Wildlife Refuge Complex U.S. Fish and Wildlife Service Honolulu HI USA

**Keywords:** bottleneck, genetic diversity, Hawaiian coot, Hawaiian gallinule, temporal genetic variation

## Abstract

The evolutionary trajectory of populations through time is influenced by the interplay of forces (biological, evolutionary, and anthropogenic) acting on the standing genetic variation. We used microsatellite and mitochondrial loci to examine the influence of population declines, of varying severity, on genetic diversity within two Hawaiian endemic waterbirds, the Hawaiian coot and Hawaiian gallinule, by comparing historical (samples collected in the late 1800s and early 1900s) and modern (collected in 2012–2013) populations. Population declines simultaneously experienced by Hawaiian coots and Hawaiian gallinules differentially shaped the evolutionary trajectory of these two populations. Within Hawaiian coot, large reductions (between −38.4% and −51.4%) in mitochondrial diversity were observed, although minimal differences were observed in the distribution of allelic and haplotypic frequencies between sampled time periods. Conversely, for Hawaiian gallinule, allelic frequencies were strongly differentiated between time periods, signatures of a genetic bottleneck were detected, and biases in means of the effective population size were observed at microsatellite loci. The strength of the decline appears to have had a greater influence on genetic diversity within Hawaiian gallinule than Hawaiian coot, coincident with the reduction in census size. These species exhibit similar life history characteristics and generation times; therefore, we hypothesize that differences in behavior and colonization history are likely playing a large role in how allelic and haplotypic frequencies are being shaped through time. Furthermore, differences in patterns of genetic diversity within Hawaiian coot and Hawaiian gallinule highlight the influence of demographic and evolutionary processes in shaping how species respond genetically to ecological stressors.

## 1 INTRODUCTION

Genetic diversity is the basis for which evolutionary forces act upon and ultimately shape the trajectory of variation within populations through time. Standing genetic variation within populations is influenced by a variety of factors: biological characteristics of the species (e.g., dispersal propensity, distribution, mating system, and generation time), human disturbance (e.g., habitat fragmentation, introduced predators, and hunting), and evolutionary forces acting although stochastic (e.g., genetic drift), deterministic (e.g., mutation), and adaptive (e.g., natural selection) processes (Amos & Hardwood, 1998). The strength at which evolutionary processes influence levels of genetic diversity is dependent, in part, on long‐term effective population sizes. Populations that are small and isolated, for example, may be predisposed to having low genetic diversity. Small populations are more likely to experience severe population fluctuations concomitant with environmental stochasticity (Melbourne & Hastings, 2008) which reduces long‐term effective population sizes and ultimately the accumulation of genetic diversity (Habel & Zachos, 2013). None of these processes are the sole driver of change; it is the interaction between evolutionary and biological forces that affect the levels of diversity within populations and species over time.

Populations occupying islands are often characterized as having reduced levels of genetic diversity relative to continental counterparts, a pattern often attributed to founder effects associated with island colonization. Among newly founded species, the genetic legacy of the founder effect may still be observed as insufficient time has passed for the species/population to overcome reductions in genetic diversity accompanying the bottleneck postcolonization (Habel & Zachos, 2013). Many island endemics, however, colonized their respective islands millions of years ago; therefore, a genetic signature of the original founding event is likely not present, and more recent processes (e.g., habitat loss) are driving observed levels of genetic variation (Stuessy, Takayama, López‐Sepúlveda, & Crawford, 2014). Although the pattern of low genetic diversity of populations occupying islands may generally hold, processes (i.e., recent effects or legacy of colonization) promoting this pattern may differ among island taxa (Habel & Zachos, 2013).

The Hawaiian Archipelago is one of the most remote island groups in the world enabling it to function like a semiclosed system prior to human arrival (500 AD); the only vertebrates to colonize it were highly vagile species (birds and bats), and its diverse avifauna can be traced to as few as 20 colonizing species (James, 1991). Similar to many Oceanic islands, however, many of the endemic species of Hawaii have gone extinct or experienced severe population declines as a result of anthropogenic influences (e.g., habitat loss/modification, introduced predators, invasive species, and disease; Blackburn, Cassey, Duncan, Evans, & Gaston, 2004). Indeed, the main Hawaiian Islands have the highest number of threatened, threatened endemic, and critical endemic restricted‐range species of the Pacific Endemic Bird Areas, many of which are likely close to extinction (BirdLife International, 2016a). Because islands were likely founded by just a few individuals, due to the remoteness of the archipelago, populations may generally harbor low levels of genetic diversity relative to mainland congeners (even prior to recent declines); this would be especially true for species that are recent colonizers. Potentially low levels of genetic diversity coupled with small population sizes may make Hawaiian endemics more susceptible to deleterious effects of environmental and demographic fluctuations. Therefore, it is important to determine standing levels of genetic variation within species that occupy the Hawaiian Islands to evaluate the impact of the population declines on genetic diversity as well as to gain insight into the ability of Hawaiian endemics to respond to stochastic processes.

**Figure 1:**
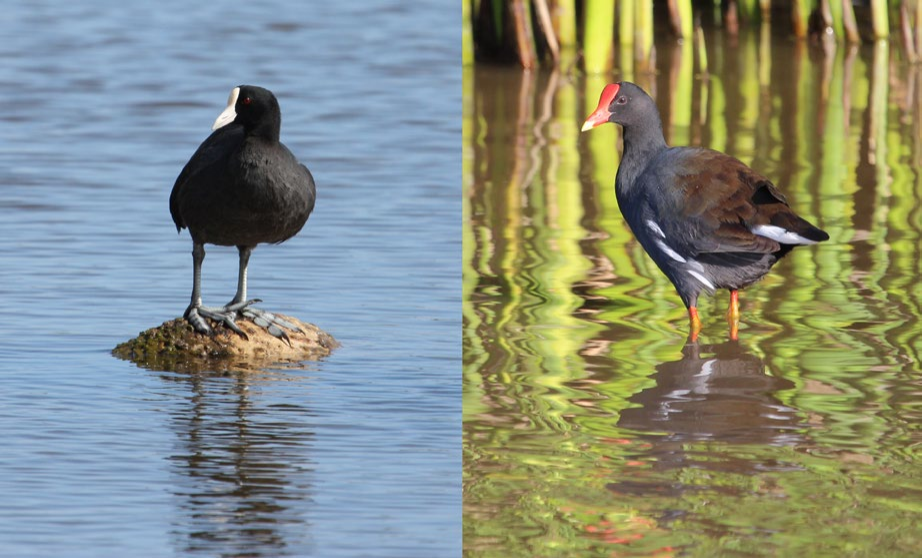
Hawaiian coot (left; *Fulica alai*) and Hawaiian gallinule (right; *Gallinula galeata sandvicensis*) in James Campbell National Wildlife Refuge, Oahu, Hawaii, USA . Photo credits: Sarah Sonsthagen (USGS) and Robert Wilson (USGS)

The Hawaiian coot (*Fulica alai*) and Hawaiian gallinule (*Gallinula galeata sandvicensis*) are endangered waterbirds endemic to the Hawaiian Archipelago (Figure 1). Both species experienced severe population declines in the early 1900s attributed to wetland loss and modification, introduced plants and predators, disease, and altered hydrology (U.S. Fish and Wildlife Service, 2011). Prior to the 1900s, both species were common and distributed across the main Hawaiian Islands (U.S. Fish and Wildlife Service, 2011), although no estimates (pre‐1900s) of population size are available. Surveys conducted in the 1950–1960s determined that census numbers were reduced to <1,000 (Hawaiian coot) and ~60 (Hawaiian gallinule) individuals throughout Hawaii (U.S. Fish and Wildlife Service, 2011). Populations started to increase in the late 1970s, likely attributable to the increase in aquaculture and the protection of wetland areas, including the establishment of several national refuges in Hawaii (U.S. Fish and Wildlife Service, 2011). The current census population size for the Hawaiian coot is 1,777 ± 310 individuals (Underwood, Silbernagle, Nishimoto, & Uyehara, 2013), and it occupies its historical range. The Hawaiian gallinule's current distribution is restricted to the islands of Oahu and Kauai, and recent surveys recorded low numbers (~400 individuals range wide), although numbers are likely underestimated as Hawaiian gallinules are secretive (Underwood et al., 2013).

Hawaiian coot and Hawaiian gallinule are hypothesized to be relatively recent colonizers to the archipelago (Fleischer & McIntosh, 2001); however, based on fossil evidence, the Hawaiian coot may have occupied the islands for a longer duration than the gallinule. A *Fulica sp*. fossil was discovered at Ulupau Head, Oahu, a formation dated to 120,000 years before present (James, 1987), and on Kauai, *F. alai* bones were identified from stratigraphic units dating up to 6,760 years before present (Burney et al., 2001). Conversely, *Gallinula chloropus* (now *G. galeata*) bones have only been found in deposits postdating human colonization (~1,500 years before present; Barber's Point, Oahu; James, 1987), although the absence of detection does not necessarily equate to lack of presence.

Many factors influence the maintenance and recovery of genetic diversity as species go through population declines. Therefore, some species within an area may be more adversely affected, while others may be more resilient in retaining genetic diversity. Here, we examine the influence of a population decline on genetic diversity simultaneously experienced by two species, the Hawaiian coot and the Hawaiian gallinule, using microsatellite genotype and mitochondrial sequence data. Specifically, we aim to examine how these species have responded genetically to recent ecological stressors (e.g., habitat loss, predation, disease). Although these two species share many life history traits, a few potentially influential demographic differences exist, such as severity of decline, effective population sizes predecline, dispersal propensity, and longevity (Bannor & Kiviat, 2002; Pratt & Brisbin, 2002). Along with providing insights into the conservation genetics of Hawaiian waterbirds, the results of this study can inform conservation planning and management strategies, especially because both species are designated as “conservation reliant,” such that they will require active management into perpetuity (Underwood et al., 2013).

## 2 METHODS

### 2.1 Samples

Specimens of Hawaiian coot (*n* = 14) and Hawaiian gallinule (*n* = 23) collected between 1893 and 1939 from Oahu, Hawaii, were obtained through museum tissue loans (Appendix 1). These samples represent the *historical* time period. Hawaiian coot (*n* = 34) and Hawaiian gallinule (*n* = 29) were trapped on James Campbell National Wildlife Refuge, Oahu, Hawaii, from 2012 to 2013. These samples represent the *modern* time period. Blood samples were collected from the brachial vein and stored in preservation buffer (Longmire et al., 1988). Hereafter, we will refer to each sampled temporal time period as a unique “population.”

### 2.2 Laboratory techniques

DNA from historical samples was extracted using a phenol–chloroform protocol and recovered with a Microcon centrifugal filter (Millipore, Massachusetts). Genomic DNA from contemporary samples was extracted using a “salting out” procedure described by Medrano, Aasen, and Sharrow (1990). Genomic DNA concentrations were quantified using fluorometry and diluted to 50 ng/ml working solutions. Genotype data were collected at 13 loci for Hawaiian coot (Fal02, Fal04, Fal08, Fal10, Fal12, Fal14, Fal16, Fal19, Gch03, Gch07, Gch12, Gch14, Sonsthagen, Wilson, & Underwood, 2014; and KiRa10, Brackett, Maley, Brumfield, & McRae, 2013) and 10 loci for Hawaiian gallinule (Fal08, Fal10, Fal12, Fal14, Fal19, Gch06, Gch12, Gch13, Gch17, and Gch19, Sonsthagen et al., 2014). Polymerase chain reaction (PCR) amplifications and thermocycler conditions followed Talbot et al. (2011). DNA extractions and PCR reactions for museum specimens were performed in a designated low‐copy laboratory to reduce the possibility of contamination from modern samples. DNA extractions and PCR reactions contained sample blanks, which were included through the data collection process. Sample blanks did not yield any product. Because DNA extracted from museum specimens may be of low quality and quantity resulting in genotyping errors (i.e., allelic dropout, and null alleles), all DNA samples from museum specimens were amplified in duplicate at all microsatellite loci, and individuals that were homozygous at loci were amplified in triplicate. In addition, 10% of the samples from James Campbell National Wildlife Refuge were extracted, amplified, and genotyped in duplicate for quality control. No inconsistencies in genotype scores were observed between replicates. Microsatellite genotype data are accessioned at the USGS Alaska Science Center data repository ( https://doi.org/10.5066/f74q7sxc).

Hawaiian coot and Hawaiian gallinule individuals were sequenced at two mitochondrial DNA (mtDNA) loci: We amplified a 824 base pair (bp) and 826 bp fragment, respectively, of control region using primer pairs CR200L (5′‐TTCATGCATGCTTTAGGG‐3′) and CR1029H (5′‐CACCAATTTCCAGRAGTCC‐3′) as well as a 752 bp and 753 bp fragment, respectively, of NADH dehydrogenase (ND) two with primer pairs ND2_224L (5′‐CTMCTACTATTCTCCAGCAC‐3′) and ND2_1003H (5′‐GGGTGATAAGGGTAGGAG‐3′). Smaller fragments of mtDNA were amplified for the historical samples using primer pairs CR200L and CR343H (5′‐GTGGRGGRGTATATTCGTG‐3′) for control region resulting in a 140 bp and 141 bp fragments in Hawaiian coot and Hawaiian gallinule, respectively, and ND2 primer pairs ND2_464L (5′‐GCCATCCTCTCAGCWGCC‐3′) and ND2_720H (5′‐GCCTGCTAGGGAKAG‐3′) resulting in a 172 bp fragment. PCR amplifications, cycle‐sequencing protocols, and postsequencing processing followed Sonsthagen, Talbot, and McCracken (2007). For quality control purposes, we extracted, amplified, and sequenced 10% of the samples in duplicate. Data generated during this study are available at doi.org/10.5066/F74Q7SXC, and sequences are accessioned in GenBank (>200 bp; MF673896–MF673904).

### 2.3 Analysis of genetic diversity

We calculated allelic richness, observed and expected heterozygosities, Hardy–Weinberg equilibrium (HWE), and linkage disequilibrium at the microsatellite loci in FSTAT version 2.9.3 (Goudet, 1995). Tests for null alleles and allelic dropout were implemented in MicroChecker (Van Oosterhout, Hutchinson, Wills, & Shipley, 2004). MtDNA sequences were trimmed to the same fragment length between historical and modern samples. Haplotype (*h*) and nucleotide (π) diversity were calculated at mtDNA loci in ARLEQUIN version 2.0 (Schneider, Roessli, & Excoffier, 2000). Fu's F_*S*_ (Fu, 1997) and Tajima's *D* (Tajima, 1989) were calculated to test the hypothesis of selective neutrality for mtDNA loci and implemented in ARLEQUIN. We applied critical significance values of 5%, which requires a *p*‐value of below .02 for Fu's F_*S*_ (Fu, 1997). An unrooted haplotype network for mtDNA loci was constructed in NETWORK version 4.613 (Fluxus Technology Ltd., 2015) using the reduced median method (Bandelt, Forster, Sykes, & Richards, 1995), to illustrate possible reticulations in the gene tree because of homoplasy or recombination.

### 2.4 Analysis of genetic structure

The degree of population genetic structure within Hawaiian coot and Hawaiian gallinule sampled pre‐ and postdecline was assessed by calculating *F*_*ST*_ and Φ_*ST*_ for microsatellite and sequence data, respectively, in ARLEQUIN, adjusting for multiple comparisons using Bonferroni correction (α = 0.05). Tamura–Nei nucleotide substitution model (Tamura & Nei, 1993) was used to calculate Φ_*ST*_. Because samples were assayed over temporal scales and sizes varied among populations, population differentiation based on χ^2^ distributions of alleles and haplotypes was also determined using GENEPOP 3.1 (Raymond & Rousett, 1995).

We used the Bayesian clustering program, STRUCTURE 2.3.2 (Hubisz, Falush, Stephens, & Pritchard, 2009; Pritchard, Stephens, & Donnelly, 2000), to assign individuals to clusters based on their microsatellite allelic frequencies and infer the occurrence of genetic structure without a priori knowledge of putative temporal period. Data were analyzed using an admixture model assuming correlated frequencies and sample location information as a prior with a 50,000 burn‐in period, 500,000 Markov chain Monte Carlo iterations, and number of possible populations (*K*) ranging from one to five; the analysis was repeated 10 times to ensure consistency across runs. We followed the method of Evanno, Regnaut, and Goudet (2005) to determine the most likely number of clusters given the data.

### 2.5 Analysis of effective population size

Contemporary effective population size (Ne) was estimated with NeESTIMATOR v2 (Do et al., 2014), using the linkage disequilibrium‐based and the molecular coancestry methods based on the microsatellite data. Molecular coancestry method examines the level of allele sharing among individuals; conversely, the linkage disequilibrium method tests for nonrandom associations formed among alleles at different loci that occur when Ne is low and genetic drift influences allelic frequencies (Luikart, Ryman, Tallmon, Schwartz, & Allendorf, 2010). Microsatellite data were analyzed using the random‐mating model, with 95% confidence limits determined by jackknifing over loci. We evaluated the effects of low‐frequency alleles on Ne estimates by excluding rare alleles (Pcrit). Variance in Ne estimates across a range of Pcrit values is suggestive of a history of gene flow and/or the presence of first‐generation dispersers, whereas stable Ne estimates across a range of Pcrit values are indicative of isolated populations (Waples & England, 2011). We estimated Ne with Pcrit values for lowest allele frequency observed ranging from 0.02 and 0.10 and without a frequency restriction.

### 2.6 Analysis of population demography

Evidence for fluctuations in historical population demography was evaluated using BOTTLENECK 1.2.02 (Cornuet & Luikart, 1996; Piry, Luikart, & Cornuet, 1999). Fluctuations in population size inferred from microsatellite data were assessed using a Wilcoxon sign‐rank test using 5,000 permutations under three models: infinite allele model (IAM), stepwise mutation model (SMM), and two‐phased model of mutation (TPM; parameters: 70% SMM, variance 9). Heterozygote deficiency relative to the number of alleles indicates recent population growth, whereas heterozygote excess indicates a recent population bottleneck (Cornuet & Luikart, 1996). It is important to note that BOTTLENECK compares heterozygote deficiency and excess relative to genetic diversity, not to HWE expectation (Cornuet & Luikart, 1996). Results were adjusted for multiple comparison using Bonferroni correction (α = 0.05).

## 3 RESULTS

### 3.1 Genetic diversity

Null alleles and allelic dropout were not detected for any loci assayed for historical and contemporary Hawaiian gallinule populations and contemporary Hawaiian coot. Within the Hawaiian coot historical population, three loci (Fal10, Gch12, and KiRa10) had evidence of null alleles. Reamplification of these loci yielded consistent genotypes, and all loci and populations were in HWE and linkage equilibrium. Therefore, we retained all loci in subsequent analyses. Indices of genetic diversity based on microsatellite loci were similar between time periods for both species (95% confidence limits overlapped), although fewer private alleles were observed within the contemporary Hawaiian gallinule (Table 1).

**Table 1:** Indices of genetic diversity along with the percent change for Hawaiian coot and Hawaiian gallinule on Oahu, Hawaii, sampled at two timescales

	Hawaiian coot			Hawaiian gallinule		
	Historical	Contemporary	% change	Historical	Contemporary	% change
Microsatellites						
No. alleles	4.9 (1.9)	5.0 (1.7)	2.0	2.6 (1.6)	2.3 (0.5)	−11.5
Allelic richness	4.6 (1.5)	4.1 (1.1)	−10.9	2.6 (1.5)	2.3 (0.4)	−11.5
Private alleles	15	16	0.0	4	1	−75.0
H_o_	55.9 (3.9)	58.9 (2.3)	5.4	42.2 (3.4)	41.0 (2.9)	−2.8
H_e_	64.9 (4.3)	61.7 (3.2)	−4.9	40.4 (6.9)	41.9 (3.7)	3.7
Ne	19.5 (0.5–71.8)	19.0 (3.1–48.6)	—	∞ (∞–∞)	5.3 (1.8–10.5)	—
*n*	13	34	—	23	29	—
mtDNA control region						
No. haplotypes	5	3	−40.0	2	1	−50.0
Private haplotypes	2	0	—	1	0	—
*h*	0.788 (0.089)	0.383 (0.098)	−51.4	0.100 (0.088)	—	—
π	0.0146 (0.0099)	0.0090 (0.0065)	−38.4	0.0007 (0.0014)	—	—
Fu's Fs	−0.3	2.2	—	−0.9	—	—
Tajima's D	1.7	0.5	—	−1.2	—	
*n*	12	31	—	20	27	—
mtDNA ND2						
No. haplotypes	2	2	0.0	3	3	0.0
Private haplotypes	0	0	—	0	0	—
*h*	0.400 (0.237)	0.220 (0.087)	−45.0	0.706 (0.042)	0.689 (0.028)	−2.4
π	0.0047 (0.0047)	0.0026 (0.0026)	−44.7	0.0055 (0.0044)	0.0053 (0.0042)	−3.6
Fu's Fs	1.0	1.4	—	0.9	1.2	—
Tajima's D	−1.0	−0.2	—	1.4	1.5	—
*n*	5	33	—	17	26	—

Descriptive statistics include the mean number of alleles and haplotypes, allelic richness, number of private alleles and haplotypes, observed and expected heterozygosity (H_o_/H_e_), effective population size (Ne) based on the molecular coancestry method, haplotype (*h*) and nucleotide diversity (π), Fu's Fs, Tajima's D, and sample size (*n*) based on 13 and 10 microsatellite loci, 140 bp of mtDNA control region, and 172 bp of mtDNA ND2. Single standard deviation is in parentheses.

**Figure 2:**
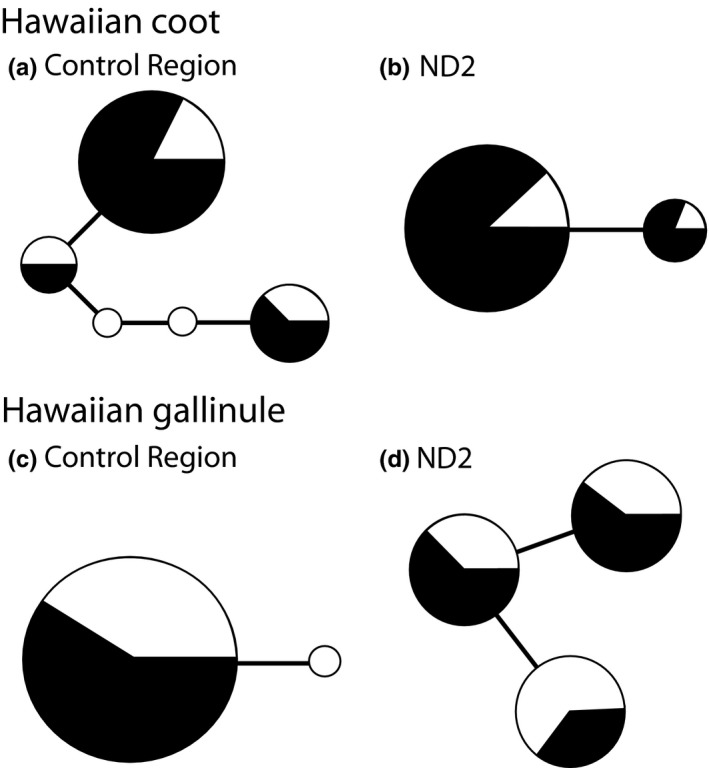
Parsimony networks illustrating relationships of mtDNA control region (a and c) and ND2 (b and d) haplotypes in Hawaiian coot and Hawaiian gallinule sampled at historical (white) and contemporary (black) timescales. The size of the circle node corresponds to the frequency of each haplotype

Within the mtDNA control region sequences, five haplotypes characterized by four variable sites were observed for Hawaiian coot, and two haplotypes characterized by a single variable site were observed for Hawaiian gallinule (Figure 2). Greater variation at ND2 was observed for Hawaiian gallinule; three haplotypes were characterized by two variable sites, whereas only two haplotypes were observed within Hawaiian coot (Figure 2). All haplotypes were retained when sequences were trimmed to a common length. A reduction in genetic diversity was observed at mtDNA for both species, although differences in indices of genetic diversity are greater for Hawaiian coot than Hawaiian gallinule (Table 1). Fewer haplotypes were observed within the mtDNA control region for the contemporary populations of Hawaiian coot and Hawaiian gallinule; further haplotype and nucleotide diversity were lower in the contemporary populations (Table 1); however, one of the two haplotypes observed in Hawaiian gallinule historical population was only found in a single individual (Figure 2). Indices of genetic diversity appear similar between timescales for Hawaiian gallinule based on ND2, although similar reductions in variation were observed for Hawaiian coot (Table 1). Tests of selective neutrality were not significant (Table 1).

### 3.2 Genetic structure

**Figure 3:**
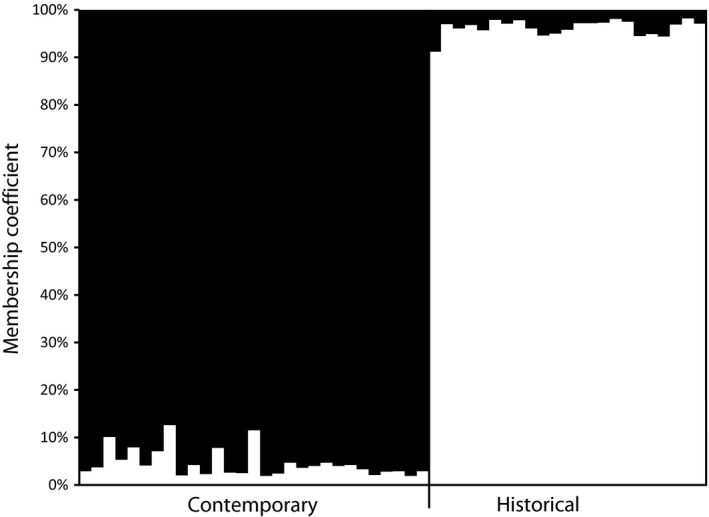
Average membership coefficient of Hawaiian gallinule individuals from sampled time periods into each of the two clusters inferred from ten microsatellite loci in STRUCTURE (Pritchard et al., 2000)

Patterns of temporal variation at allelic and haplotypic frequencies varied between species. A strong signature of genetic structure was observed within Hawaiian gallinule as frequency differences among microsatellite loci were detected across all three metrics (*F*_*ST*_, *R*_*ST*_, and χ^2^; Table 2) and was also uncovered in STRUCTURE (K = 2, ΔK = 162.4, *r* = .44; Figure 3). Conversely, only a weak signature of genetic structure was detected in Hawaiian coot based on microsatellite data; differences in allelic distributions (χ^2^) were observed; however, no structure was detected based on *F*_*ST*_ and *R*_*ST*_ (Table 2) nor was genetic partitioning uncovered in STRUCTURE (K = 1, LnP|K = −1548.9; K = 2, LnP|K = −1567.5). Frequency differences were detected in mtDNA control region haplotypic data based on *F*_*ST*_ and χ^2^ distributions between historical and contemporary Hawaiian coot populations (Table 2). No differences were observed at mtDNA control region within Hawaiian gallinule between time periods, as only two haplotypes were observed and one haplotype represented by a single individual (Figure 2).

**Table 2:** Estimates of genetic differentiation (*F*_*ST*_, *R*_*ST*_, χ^2^, and Φ_*ST*_) calculated from 13 and 10 microsatellite loci, respectively, 140 bp of mtDNA control region, and 172 bp of mtDNA ND2 between Hawaiian coot and Hawaiian gallinule sampled at two time periods. Significant comparisons (α = 0.05) are in bold text and marked with an asterisk

	Microsatellites			mtDNA control region			mtDNA ND2		
	*F*_*ST*_	*R*_*ST*_	χ^2^	*F*_*ST*_	Φ_*ST*_	χ^2^	*F*_*ST*_	Φ_*ST*_	χ^2^
Hawaiian coot	0.014	−0.001	68.7*	0.095*	0.096	11.3*	−0.098	−0.098	1.0
Hawaiian gallinule	0.182*	0.073*	∞*	0.015	0.015	3.6	−0.050	−0.050	0.2

### 3.3 Effective population size

**Figure 4:**
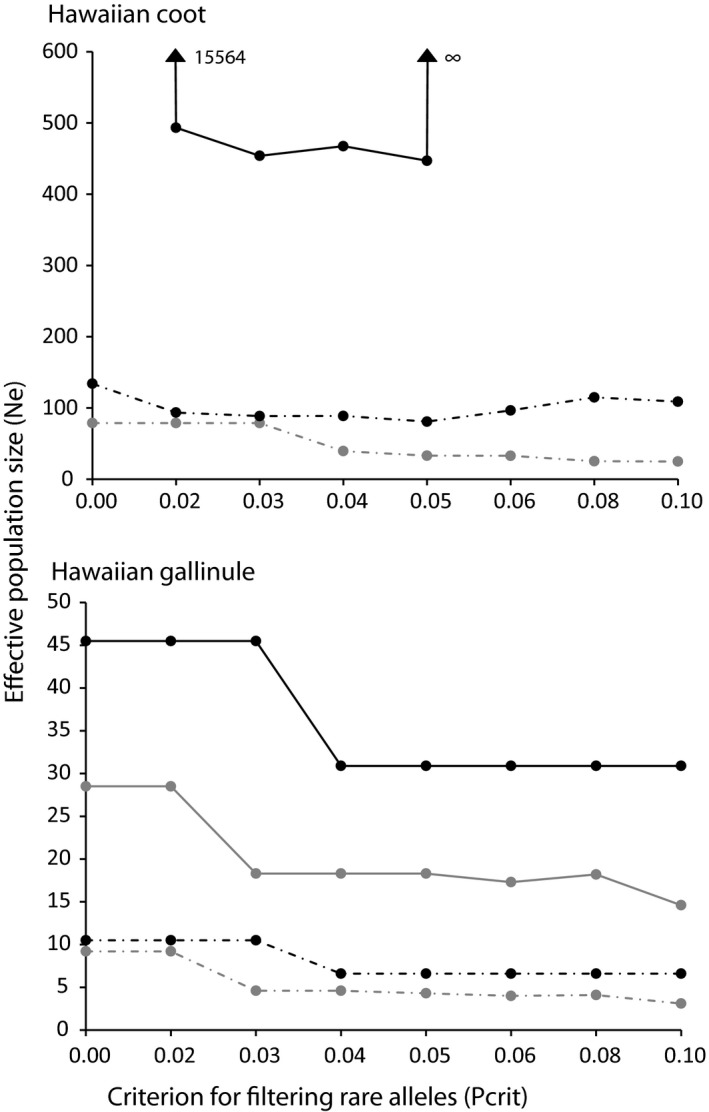
Effective population size (Ne) estimates as a function of excluding of rare alleles (Pcrit) in Hawaiian coot and Hawaiian gallinule sampled at historical (shown in gray) and contemporary (shown in black) timescales, implemented in NeESTIMATOR v2 (Do et al., 2014). The solid line represents point estimates of Ne, and dash lines are associated lower 5% confidence limits. Upper 95% confidence limits and Ne for historical Hawaiian coot are ∞ for all estimates. Values of point estimates that exceed the range of the *y*‐axis are noted on the graph

Genetic signatures of a reduction in Ne were not observed in either Hawaiian coot or Hawaiian gallinule based on the linkage disequilibrium method, as 95% confidence limits overlapped between sampling periods and the upper bound was infinity (Figure 4). Variation in Ne estimates across Pcrit values was observed for contemporary Hawaiian coot individuals, indicative of past gene flow affecting Ne estimates. In contrast, Ne estimates were similar across Pcrit values in Hawaiian gallinule. While Ne estimates based on the molecular coancestry method overlapped for both Hawaiian coot and Hawaiian gallinule, the contemporary Hawaiian gallinule estimates were considerably smaller with narrow confidence intervals (Table 1).

### 3.4 Population demography

Fluctuations in population demography were observed within the contemporary Hawaiian gallinule timeframe; significant heterozygote excess was detected at all mutation models (IAM, SMM, and TPM; *p* < .001) suggestive of population decline. The other timeframes were in mutation drift equilibrium.

## 4 DISCUSSION

The evolutionary trajectories of the Hawaiian coot and Hawaiian gallinule were differentially shaped by the population declines of the early 1900s. Within Hawaiian coots, large reductions (between −38.4% and −51.4%; Table 1) in mitochondrial diversity were observed, although minimal differences (χ^2^ = 68.7, *p* < .001; *F*_*ST*_ and *R*_*ST*_, *p *>* *.05; Table 2) were observed in the distribution of allelic and haplotypic frequencies across timescales. Conversely, for Hawaiian gallinules, allelic frequencies were strongly differentiated between time periods, signatures of a genetic bottleneck were detected, and biases in the means of the effective population size were observed at microsatellite loci. Researchers have invoked several hypotheses for the lack of observed temporal shifts in genetic diversity within species that have experienced declines. Generally, when reductions in genetic diversity are observed, those populations are often reduced to just a few individuals (e.g., Bouzat, Lewin, & Paige, 1998; Groombridge, Jones, Bruford, & Nichols, 2000). However, long‐lived species can reduce the effective time spent experiencing a bottleneck thus enabling the retention of genetic diversity over short timescales (e.g., Hailer et al., 2006; Johnson, Gilbert, Virani, Asim, & Mindell, 2008; White et al., 2014). It is important to note, our ability to detect a recent bottleneck is dependent on the level of genetic diversity pre‐ and postdecline as ancient reductions in genetic diversity may mask recent declines (e.g., Dussex, Rawlence, & Robertson, 2015). While the strength of the decline appears to be greater within Hawaiian gallinule than Hawaiian coot, coincident with the reduction in census size, these species exhibit similar life history characteristics and generation times (7 and 6 years, respectively; BirdLife International 2016b,c). Therefore, we hypothesize that differences in behavior and colonization history are likely playing a large role in how allelic and haplotypic frequencies within these species are being shaped through time.

Hawaiian coots and Hawaiian gallinules are capable of dispersing long distances, as evidenced by their successful colonization of the Hawaiian Archipelago. Postcolonization, however, dispersal propensity appears to have changed. Current behavior for the Hawaiian gallinule is described as highly sedentary (Bannor & Kiviat, 2002; JGU unpublished data), whereas mark/resight data for Hawaiian coot suggest there is some interisland movement (JGU unpublished data). It is difficult to distinguish whether changes in dispersal behavior are an artifact of colonization or recent anthropogenic‐caused declines. Regardless, reduction in gene flow among remnant patches will lead to loss of genetic diversity through drift, as drift is inversely related to the effective population size (1/2Ne; Frankham, Ballou, & Briscoe, 2010). Movement of individuals among islands may provide an avenue for dispersal and in turn greater retention of genetic diversity through increased effective population size thereby reducing the effects of drift (Frankham et al., 2010; Jangjoo, Matter, Roland, & Keyghobadi, 2016). Within the more sedentary Hawaiian gallinule, population recovery was likely the result of recruitment within remnant wetland patches, and therefore, the survivors of the decline changed the trajectory of neutral genetic variation as evidenced by significant structuring (Table 2, Figure 3) and reduction of Ne between sample periods (based on molecular coancestry; Table 1). The Hawaiian gallinule remains extirpated from islands in its former range in Hawaii, further supporting the hypothesis that dispersal remains restricted and recovery likely occurred through recruitment within patches rather than immigration among patches. Conversely, within Hawaiian coot, recovery was likely the result of recruitment within and dispersal among wetlands, which is supported by fluctuations in Ne estimates across Pcrit values (Figure 4). Assuming interisland movement resulted in gene flow, immigration among islands likely counteracted the effects of genetic drift and homogenized neutral genetic variation while maintaining the predecline level genetic diversity through the demographic bottleneck (e.g., Jangjoo et al., 2016; Keller et al., 2001; McEachern, Van Vuren, Floyd, May, & Eadie, 2011).

Levels of genetic diversity observed for Hawaiian gallinule are, at least in part, a legacy of the initial colonization event. Across all measures of genetic diversity, Hawaiian coot is more diverse than the Hawaiian gallinule. As noted earlier, both species are presumed to be recent colonizers to the Hawaiian Islands. The presence of fossils for Hawaiian coot and lack of remains of Hawaiian gallinule in older deposits (<1,500 years before present) provide anecdotal evidence that Hawaiian coot has occupied the Hawaiian Archipelago for a longer duration and therefore has had more time to overcome the initial reduction in genetic diversity in the wake of colonization. Among the same 48 microsatellite loci screened for variability, genetic diversity is ~48% less within Hawaiian gallinule (1.44 alleles/locus, 1–3 alleles observed, 31 loci monomorphic) than Hawaiian coot (2.75 alleles/locus, 1–7 alleles observed, 15 monomorphic, based on 10 individuals; Sonsthagen et al., 2014; SAS unpublished data). Ancient reductions in genetic diversity have been postulated to limit the signature of recent population declines (e.g., Dussex et al., 2015; Taylor & Jamieson, 2008). Given that genetic diversity was likely already low prior to decline for Hawaiian gallinule, the observation of genetic structuring between sample time periods is even more noteworthy because genetic drift had little variation on which to act indicating that the individuals that survived the bottleneck had a strong influence on the evolutionary trajectory of the Oahu population.

This study highlights the importance of species behavior and past evolutionary forces in shaping how species respond genetically to ecological stressors. Factors and time frame leading to the population decline were similar for the Hawaiian coot and Hawaiian gallinule: reduction in coastal wetlands, nonnative mammalian predators and plants, avian disease, and altered hydrology. Both were historically abundant with severe reductions in census size (<1,000 Hawaiian coot and ~60 Hawaiian gallinule in the mid‐1900s) and have similar life history characteristics. Furthermore, because these species cooccur where their distributions overlap, management strategies were implemented to conserve these species simultaneously. Yet despite these similarities, allelic frequencies differed markedly pre‐ and postdecline within Hawaiian gallinule, and only minimal differences were observed within Hawaiian coot. These findings illustrate the importance of considering how demographic (e.g., dispersal) and evolutionary processes (e.g., bottleneck effects) influence population recovery when planning and implementing conservation programs, especially for endemic species that likely harbor reduced levels of genetic variation relative to their mainland counterparts. In light of these results, Hawaiian gallinule may be at increased risk of genetic endangerment relative to its ecological equivalent, the Hawaiian coot. Although indices of genetic diversity are not reduced (confidence intervals overlap), allelic frequencies have changed indicating genetic drift is strongly influencing genetic variation within the Hawaiian gallinule on Oahu. Continued persistence in isolation, coupled with low genetic diversity, could result in the continued reduction of genetic diversity through genetic drift making the Hawaiian gallinule more vulnerable to stochastic processes and ultimately extirpation.

## AUTHOR CONTRIBUTIONS

All authors were involved with the concept and design; JGU collected the genetic samples; REW and SAS collected the genetic data and conducted the analyses; all authors were involved in interpretation of the results; SAS led the writing; and all authors were involved in the writing process. All authors approved of the final draft of the manuscript.

## CONFLICT OF INTEREST

None declared.

## APPENDIX 1 List of museum voucher specimens used in this study along with collection year

### 1.1

**Table:**

Hawaiian coot		Hawaiian gallinule	
Catalog no.	Collection year	Catalog no.	Collection year
AMNH 193232	1907	AMNH 546980	1893
AMNH 193233	1907	AMNH 546981	1893
ANSP 33672	1835	AMNH 546982	1893
BBM 4544	1907	AMNH 546983	1893
BBM 4545	1907	AMNH 546984	1893
BBM 4547	1907	AMNH 546985	1893
LSUMZ 81484	1939	AMNH 546986	1893
MCZ 55659	1895	BBM 4542	1907
MCZ 55660	1895	BBM 6307	1936
MCZ 80938	1895	BBM 6449	1938
MCZ 80939	1895	MCZ 39245	1894
MCZ 115018	1895	MCZ 55648	1895
MCZ 115019	1895	MCZ 55650	1895
		MCZ 55651	1895
		MCZ 55652	1895
		MCZ 55654	1895
		MCZ 55655	1895
		MCZ 55656	1895
		MCZ 55657	1895
		MCZ 239942	1894
		MVZ 7037	1903
		MVZ 7040	1909
		USNM 301112	1923

AMNH, American Museum of Natural History; ANSP, Academy of Natural Sciences of Philadelphia; BBM, Bernice Pauahi Bishop Museum; LSUMZ, Louisiana State University Museum of Natural Science; MCZ, Harvard University Museum of Comparative Zoology; MVZ, University of California Berkeley Museum of Vertebrate Zoology; and USNM, National Museum of Natural History.

## References

[ece33530-bib-0001] Amos, W. , & Hardwood, J. (1998). Factors affecting levels of genetic diversity in natural populations. Philosophical Transactions of the Royal Society of London Series B, 353, 177–186. 9533122 10.1098/rstb.1998.0200 PMC1692205

[ece33530-bib-0002] Bandelt, H. J. , Forster, P. , Sykes, B. C. , & Richards, M. B. (1995). Mitochondrial portraits of human populations. Genetics, 141, 743–753. 8647407 10.1093/genetics/141.2.743 PMC1206770

[ece33530-bib-0003] Bannor, B. K. , & Kiviat, E. (2002). Common Gallinule (Gallinula galeata), The Birds of North America (P. G. Rodewald, Ed.). Ithaca: Cornell Lab of Ornithology. Retrieved from the Birds of North America: https://birdsna.org/Species-Account/bna/species/comgal1 https://doi.org/10.2173/bna.685

[ece33530-bib-0004] BirdLife International . (2016a). Endemic Bird Area factsheet: Central Hawaiian Islands. Retrieved from http://www.birdlife.org

[ece33530-bib-0005] BirdLife International . (2016b). Fulica alai. The IUCN Red List of Threatened Species 2016: e.T22692920A93374177. Retrieved from https://doi.org/10.2305/iucn.uk.2016-3.rlts.t22692920a93374177.en

[ece33530-bib-0006] BirdLife International . (2016c). Gallinula galeata. The IUCN Red List of Threatened Species 2016: e.T62120280A95189182. Retrieved from https://doi.org/10.2305/iucn.uk.2016-3.rlts.t62120280a95189182.en

[ece33530-bib-0007] Blackburn, T. M. , Cassey, P. , Duncan, R. P. , Evans, K. L. , & Gaston, K. J. (2004). Avian extinction and mammalian introductions on Oceanic islands. Science, 305, 1955–1958. 15448269 10.1126/science.1101617

[ece33530-bib-0008] Bouzat, J. L. , Lewin, H. A. , & Paige, K. N. (1998). The ghost of genetic diversity past: Historical DNA analysis of the greater prairie chicken. American Naturalist, 152, 1–6. 10.1086/286145 18811397

[ece33530-bib-0009] Brackett, C. L. , Maley, J. M. , Brumfield, R. T. , & McRae, S. B. (2013). Characterization of microsatellite loci for a threatened species, the King Rail, *Rallus elegans*, using a next‐generation sequencing protocol. Conservation Genetics Resources, 5, 1189–1191.

[ece33530-bib-0010] Burney, D. A. , James, H. F. , Pigott Burney, L. , Olson, S. L. , Kikuchi, W. , Wagner, W. L. , … Nishek, R. (2001). Fossil evidence for a diverse biota from Kaua'i and its transformation since human arrival. Ecological Monographs, 71, 615–641.

[ece33530-bib-0011] Cornuet, J. M. , & Luikart, G. (1996). Description and power analysis of two tests for detecting recent population bottlenecks from allele frequency data. Genetics, 144, 2001–2014. 8978083 10.1093/genetics/144.4.2001 PMC1207747

[ece33530-bib-0012] Do, C. , Waples, R. S. , Peel, D. , Macbeth, G. M. , Tillett, B. J. , & Ovenden, J. R. (2014). NeEstimator v2: Re‐implementation of software for the estimation of contemporary effective population size (Ne) from genetic data. Molecular Ecology Resources, 14, 209–214. 23992227 10.1111/1755-0998.12157

[ece33530-bib-0013] Dussex, N. , Rawlence, N. J. , & Robertson, B. C. (2015). Ancient and contemporary DNA reveal a pre‐human decline but no population bottleneck associated with recent human persecution in the Kea (*Nestor notabilis*). PLoS ONE, 1092, e0118522. 10.1371/journal.pone.0118522 PMC4342260 25719752

[ece33530-bib-0014] Evanno, G. , Regnaut, S. , & Goudet, J. (2005). Detecting the number of clusters of individuals using the software STRUCTURE: A simulation study. Molecular Ecology, 14, 2611–2620. 15969739 10.1111/j.1365-294X.2005.02553.x

[ece33530-bib-0015] Fleischer, R. C. , & McIntosh, C. E. (2001). Molecular systematics and biogeography of the Hawaiian avifauna. Studies in Avian Biology, 22, 51–60.

[ece33530-bib-0016] Fluxus Technology Ltd . (2015). NETWORK 4.6.1.3. Retrieved from www.fluxus‐engineering.com

[ece33530-bib-0017] Frankham, R. , Ballou, J. D. , & Briscoe, D. A. (2010). Introduction to conservation genetics, 2nd ed. Cambridge: Cambridge University Press.

[ece33530-bib-0018] Fu, Y. X. (1997). Statistical tests of neutrality of mutations against population growth, hitchhiking and background selections. Genetics, 147, 915–925. 9335623 10.1093/genetics/147.2.915 PMC1208208

[ece33530-bib-0019] Goudet, J. (1995). FSTAT (vers. 1.2): A computer program to calculate F‐statistics. Journal of Heredity, 86, 485–486.

[ece33530-bib-0020] Groombridge, J. J. , Jones, G. , Bruford, M. W. , & Nichols, R. A. (2000). Conservation biology – ‘Ghost’ alleles of the Mauritius kestrel. Nature, 403, 616. 10688188 10.1038/35001148

[ece33530-bib-0021] Habel, J. N. , & Zachos, F. E. (2013). Past population history versus recent population decline – Founder effects in island species and their genetic signatures. Journal of Biogeography, 40, 206–207.

[ece33530-bib-0022] Hailer, F. , Helander, B. , Folkestad, A. O. , Ganusevich, S. A. , Garstad, S. , Hauff, P. , … Ellegren, H. (2006). Bottlenecked but long‐lived: High genetic diversity retained in white‐tailed eagles upon recovery from population decline. Biology Letters, 2, 316–319. 17148392 10.1098/rsbl.2006.0453 PMC1618921

[ece33530-bib-0023] Hubisz, M. A. , Falush, D. , Stephens, M. , & Pritchard, J. K. (2009). Inferring weak population structure with the assistance of sample group information. Molecular Ecology Resources, 9, 1322–1332. 21564903 10.1111/j.1755-0998.2009.02591.x PMC3518025

[ece33530-bib-0024] James, H. F. (1987). A late Pleistocene avifauna from the island of Oahu, Hawaiian Islands. Documents des Laboratoires de Geologie de Lyon, 99, 221–230.

[ece33530-bib-0025] James, H. F. (1991). The contribution of fossils to knowledge of Hawaiian birds. Acta XX Congressus Internationalis Ornithologici, 1, 420–424.

[ece33530-bib-0026] Jangjoo, M. , Matter, S. F. , Roland, J. , & Keyghobadi, N. (2016). Connectivity rescues genetic diversity after a demographic bottleneck in a butterfly population network. Proceedings of the National Academy of Sciences of the United States of America, 113, 10914–10919. 27621433 10.1073/pnas.1600865113 PMC5047165

[ece33530-bib-0027] Johnson, J. A. , Gilbert, M. , Virani, M. Z. , Asim, M. , & Mindell, D. P. (2008). Temporal genetic analysis of the critically endangered oriental white‐backed vulture in Pakistan. Biological Conservation, 141, 2403–2409.

[ece33530-bib-0028] Keller, L. F. , Jeffery, K. J. , Arcese, P. , Beaumont, M. A. , Hochachka, W. M. , Smith, J. N. M. , & Bruford, M. W. (2001). Immigration and the ephemerality of a natural population bottleneck: Evidence from molecular markers. Proceedings of the Royal Society B, 268, 1387–1394. 11429139 10.1098/rspb.2001.1607 PMC1088753

[ece33530-bib-0029] Longmire, J. L. , Lewis, A. K. , Brown, N. C. , Buckingham, J. M. , Clark, L. M. , Jones, M. D. , … Moyzis, R. K. (1988). Isolation and molecular characterization of a highly polymorphic centromeric tandem repeat in the family Falconidae. Genomics, 2, 14–24. 3384438 10.1016/0888-7543(88)90104-8

[ece33530-bib-0030] Luikart, G. , Ryman, N. , Tallmon, D. A. , Schwartz, M. K. , & Allendorf, F. W. (2010). Estimation of census and effective population sizes: The increasing usefulness of DNA‐based approaches. Conservation Genetics, 11, 355–373.

[ece33530-bib-0031] McEachern, M. B. , Van Vuren, D. H. , Floyd, C. H. , May, B. , & Eadie, J. M. (2011). Bottlenecks and rescue effects in a fluctuating population of golden‐mantled ground squirrels (*Spermophilus lateralis*). Conservation Genetics, 12, 285–296.

[ece33530-bib-0032] Medrano, J. F. , Aasen, E. , & Sharrow, L. (1990). DNA extraction from nucleated red blood cells. BioTechniques, 8, 43. 2182076

[ece33530-bib-0033] Melbourne, B. A. , & Hastings, A. (2008). Extinction risk depends strongly on factors contributing to stochasticity. Nature, 454, 100–103. 18596809 10.1038/nature06922

[ece33530-bib-0034] Piry, S. , Luikart, G. , & Cornuet, J. M. (1999). BOTTLENECK: A computer program for detecting recent reductions in the effective population size using allele frequency data. Journal of Heredity, 90, 502–503.

[ece33530-bib-0035] Pratt, H. D. , Brisbin Jr, I. L. (2002). Hawaiian Coot (Fulica alai), The Birds of North America (P. G. Rodewald, Ed.). Ithaca: Cornell Lab of Ornithology; Retrieved from the Birds of North America: https://birdsna.org/Species-Account/bna/species/hawcoo https://doi.org/10.2173/bna.697b

[ece33530-bib-0036] Pritchard, J. K. , Stephens, M. , & Donnelly, P. (2000). Inference of population structure from multilocus genotype data. Genetics, 155, 945–959. 10835412 10.1093/genetics/155.2.945 PMC1461096

[ece33530-bib-0037] Raymond, M. , & Rousett, F. (1995). GENEPOP (version 1.2): Population genetics software for exact tests and ecumenicism. Journal of Heredity, 86, 248–249.

[ece33530-bib-0038] Schneider, S. , Roessli, D. , & Excoffier, L. (2000). ARLEQUIN version 2.0: A software for population genetic data analysis. Geneva: Genetics and Biometry Laboratory, University of Geneva.

[ece33530-bib-0039] Sonsthagen, S. A. , Talbot, S. L. , & McCracken, K. G. (2007). Genetic characterization of common eiders (*Somateria mollissima*) breeding on the Yukon‐Kuskokwim Delta, Alaska. Condor, 109, 879–894.

[ece33530-bib-0040] Sonsthagen, S. A. , Wilson, R. E. , & Underwood, J. G. (2014). Development and characterization of microsatellite markers for the Hawaiian coot, *Fulica alai*, and Hawaiian gallinule, *Gallinula galeata sandvicensis*, through next‐generation sequencing. Conservation Genetics Resources, 6, 765–767.

[ece33530-bib-0041] Stuessy, T. F. , Takayama, K. , López‐Sepúlveda, P. , & Crawford, D. J. (2014). Interpretation of patterns of genetic variation in endemic plant species of oceanic islands. Botanical Journal of the Linnean Society, 174, 276–288. 26074627 10.1111/boj.12088 PMC4459035

[ece33530-bib-0042] Tajima, F. (1989). The effect of change in population size on DNA polymorphism. Genetics, 123, 597–601. 2599369 10.1093/genetics/123.3.597 PMC1203832

[ece33530-bib-0043] Talbot, S. L. , Palmer, A. G. , Sage, G. K. , Sonsthagen, S. A. , Swem, T. , Brimm, D. J. , & White, C. M. (2011). Lack of genetic polymorphism among peregrine falcons *Falco peregrinus* of Fiji. Journal of Avian Biology, 42, 415–428.

[ece33530-bib-0044] Tamura, K. , & Nei, M. (1993). Estimation of the number of nucleotide substitutions in the control region of mitochondrial DNA in humans and chimpanzees. Molecular Biology and Evolution, 10, 512–526. 8336541 10.1093/oxfordjournals.molbev.a040023

[ece33530-bib-0045] Taylor, S. S. , & Jamieson, I. G. (2008). No evidence for loss of genetic variation following sequencing translocations in extant populations of a genetically depauperate species. Molecular Ecology, 17, 545–556. 17986194 10.1111/j.1365-294X.2007.03591.x

[ece33530-bib-0046] Underwood, J. G. , Silbernagle, M. , Nishimoto, M. , & Uyehara, K. (2013). Managing conservation reliant species: Hawai'i's endangered endemic waterbirds. PLoS ONE, 8(6), e67872 https://doi.org/10.1371/journal.pone.0067872 23825687 10.1371/journal.pone.0067872 PMC3692473

[ece33530-bib-0047] U.S. Fish and Wildlife Service (2011). Recovery plan for Hawaiian waterbirds, second revision. Oregon: U.S. Fish and Wildlife Service, Portland. Xx+233 pp.

[ece33530-bib-0048] Van Oosterhout, C. , Hutchinson, W. F. , Wills, D. P. M. , & Shipley, P. (2004). Micro‐checker: Software for identifying and correcting genotyping errors in microsatellite data. Molecular Ecology Notes, 4, 535–538. https://doi.org/10.1111/j.1471-8286.2004.00684.x

[ece33530-bib-0049] Waples, R. S. , & England, P. R. (2011). Estimating contemporary effective population size on the basis of linkage disequilibrium in the face of migration. Genetics, 189, 633–644. 21840864 10.1534/genetics.111.132233 PMC3189803

[ece33530-bib-0050] White, N. E. , Bunce, M. , Mawson, P. R. , Dawson, R. , Saunders, D. A. , & Allentoft, M. E. (2014). Identifying conservation units after large‐scale land clearing: A spatio‐temporal molecular survey of endangered white‐tailed black cockatoos (*Calyptorhynchus* spp.). Diversity and Distributions, 20, 1208–1220.

